# On the Limits of Diversity

**DOI:** 10.3389/fgene.2012.00136

**Published:** 2012-07-26

**Authors:** Yann C. Klimentidis

**Affiliations:** ^1^Section on Statistical Genetics, Department of Biostatistics, University of Alabama at BirminghamBirmingham, AL, USA

A sense of wonder at life and evolution on our planet is not lost on George McGhee, author of *Convergent Evolution*. This wonder is tempered, however, by the realization that the diversity of life on our planet, and possibly other planets, is constrained by several forces. This major theme is indicated in the subtitle “*Limited Forms Most Beautiful*,” a play on the words of Charles Darwin, “… endless forms most beautiful…,” in *On the Origin of Species*. Indeed, convergent evolution results from the finite number of ecological niches on our planet, each of which is exploited by many organisms that share a common origin.

The book is organized into eight chapters beginning with a clarification of the minor difference between convergent vs. parallel evolution, and of the idea of evolutionary constraint. The next two chapters focus on various examples of convergent form and function in animals and in plants. For each example of convergent evolution, McGhee provides a table of the various phylogenetic lineages that have converged on the same trait. The traits covered range from the various forms of locomotion, prey capture, predator avoidance, organ systems, to the consideration of hypothetical body plans such as the mythical centaur.

Examples of convergent evolution abound in plants. We learn that arborescence (tree structure), as an adaptation to life on land, independently arose in nine different lineages, as did leaves, which increase the surface area for photosynthesizing food. Interestingly, eye spots have arisen 11 times in plants (algae). Other examples of convergent evolution in plants include anti-herbivore, anti-dehydration, and fertilization systems, which developed independently in many plant lineages.

The next chapters focus on less known examples of convergent evolution at “higher” and “lower” levels of organization: ecosystems, molecules, and minds. McGhee discusses convergent ecosystems in isolated geographical regions such as New Zealand and Madagascar. Communities of organisms converge on these ecosystems much as they do in other parts of the world. At lower levels of organization, DNA sequence also clearly exhibits convergence such as when identical mutations arise in different lineages in genes related to visual pigments in primates, digestive enzymes, or pigmentation. For example, many mutations have independently arisen in the *OCA2* and *MC1R* genes in various lineages resulting in different pigmentation traits.

Biologists, ecologists, and anthropologists, among others, may rejoice in learning that their “favorite” species or ecosystems are not so unique after all, and that similar solutions have been developed many times before. The chapter on convergent minds is particularly fascinating in this context of human traits. As an anthropologist, I was especially intrigued to learn that the many elaborate behaviors comprising crop agriculture and animal husbandry that we might perceive as uniquely human have been developed many times and long ago by several sets of organisms. Other behaviors that show convergence across many organisms are mourning, group hunting, self-awareness, and architectural endeavors.

In the last two chapters, McGhee extends our knowledge gained about convergent evolution on Earth to consider the possibility of life elsewhere. Is four-base DNA the only possible genetic code? Are right-coiled amino acids possible? Could silicon instead of carbon be the building block of molecules? Although this might appear to involve overly speculative debates, McGhee discusses the evidence for the above issues with the available empirical information, and in the light of what we have learned from convergent evolution on our planet.

In the last chapter of the book, McGhee delves into some of the philosophical debates concerning the path of evolution on our planet. Specifically, McGhee covers the debate over the question: “Is evolution predictable, preordained, and inevitable?” This is based on a question posed by Stephen J. Gould which essentially asks whether the path taken by evolution on our planet since the Big Bang would be any different if the process were to be repeated. McGhee appears to argue that there is evidence showing that evolution is indeed predictable and inevitable to some extent.

This book is a fascinating compendium that at the same time documents the diversity of life and illustrates how within this diversity there exist a limited number of paths through which to exploit the available ecological niches on our planet. As there are many angles from which to examine convergent evolution, the author is not able to go into great detail on any one subject. One area in which new insights into convergent evolution are quickly being generated is the genetic basis of convergent evolution, perhaps the subject of its’ own book in the future.

*Convergent Evolution* appears to be one of very few books that focus specifically on the topic of convergent evolution. It is very well referenced and extensively documents specific examples of convergent evolution. Throughout the book, one is amazed at the author's vast knowledge about a wide range of living forms, past and present. Although it appears to focus on a specific part of evolution, it reaches into many other areas of evolution, biology, and science, and is admirably adventurous in its use of thought experiments (aided by empirical data) that consider the existence of other life forms, life on other planets, and whether life on our planet could have gone down a radically different path if the entire process were to be repeated. I would highly recommend this book for any intermediate or advanced course related to evolution, or to any person with an interest in evolution, biology, or life on our planet.

